# Bilateral multiple trigger fingers in a pediatric patient: A rare case report

**DOI:** 10.1016/j.ijscr.2025.111575

**Published:** 2025-06-27

**Authors:** Maha D. Hanawi, Rakan H. Alelyani, Kayan F. Alotaibi, Abdulrahman I. Alhadlaq, Ovais Habib, Bandar A. Alaithan

**Affiliations:** a Department of Plastic & Reconstructive Surgery, King Fahad Medical City, Riyadh, Saudi Arabia; b Department of Plastic & Reconstructive Surgery, Ministry of National Guard, Health Affairs, Riyadh, Saudi Arabia; c College of Medicine, Princess Nourah Bint Abdulrhman University, Riyadh, Saudi Arabia; d Department of Plastic & Reconstructive Surgery, Security Forces Hospital, Riyadh, Saudi Arabia

**Keywords:** Trigger finger, Bilateral hands, Surgical management, Conservative approach, Children, Case report

## Abstract

**Introduction and importance:**

Trigger finger, or stenosing flexor tenosynovitis, is a condition characterized by a mismatch between the flexor tendon and its surrounding sheath at the A1 pulley, which facilitates smooth movement.

We are presenting a rare case of bilateral index and ring fingers triggering in a 5-year-old boy, which necessitates surgical release, and a brief literature review.

**Case presentation:**

We report a case of 5-year-old boy who presented to our clinic with his mother with 1 year history of bilateral hands multiple fingers triggering. Mother reported on and off bilateral index and ring fingers triggering that is passively correctable. Patient examined and found to have nodules over the index and ring fingers with active triggering. After exclusion of underlying causes, patient was referred initially for physiotherapy and have been followed up with no improvement. Surgical release of trigger fingers was planned as staged procedures starting with the left hand then the right. Patient followed up post-op, has full range of motion of his fingers with no issues.

**Clinical discussion:**

We believe that multiple trigger fingers in bilateral hands of pediatric cases are rare, and they may be associated with some underlying diseases that should be addressed and managed before any surgical management.

**Conclusion:**

Unlike trigger thumb which is very common in pediatrics, trigger finger is considered rare, and its bilateral hands involvement is the rarest. The pathophysiology of trigger finger in pediatrics differs that in adults, so detailed history, physical exam, and work-up must be done before any surgical intervention plan as it may be resolved spontaneously after managing the underlying cause.

## Highlights

• Bilateral multiple trigger fingers in children are rare and a few cases have been reported.
• A 5-year-old boy with bilateral index and ring finger triggering failed conservative management and had surgery.
• The case emphasizes the importance of excluding any underlying conditions such as mucopolysaccharidosis before surgery.
• Partial A2 pulley release in addition to A1to achieve complete tendon gliding, exhibiting surgical variations.
• This report adds to the limited literature on pediatric trigger fingers and follows the SCARE 2025 guidelines.

## 1 Introduction

Trigger finger, which is also known as stenosing flexor tenosynovitis, is a condition where a mismatch occurs between the flexor tendon and the sheath surrounding it at the A1 pulley which permits its smooth passage. That process prevents the smooth gliding of the flexor tendon through the sheath and leads to various stages of triggering ranging from a palpable nodule (Notta's nodule) to a fixed finger deformity [1].

Trigger thumb is a common presentation in pediatric patients represents 10 times more common than trigger finger [1]. Trigger finger is rare in children accounts for only 0.005 % and bilateral involvement of multiple fingers is considered the rarest with only 4 case reports documented in the literature [2].

The pathophysiology of trigger fingers in pediatrics population differs than in adults [3]. The nature cause of trigger fingers in infants is still debated either is it congenital or acquired. Trauma, anatomical abnormalities, infections and underlying disorders like diabetes and mucopolysaccharidosis have been associated with trigger fingers in pediatrics [4].

It is classified into 4 grades; grade I has only a palpable hard nodule at or distal to the A1 pulley, stage II represents snapping while actively extend the finger with complete range of motion, grade III finger triggering in flexion with limited range of motion that must be corrected passively, stage IV explains the fixed finger deformity that is not corrected with passive extension [5].

No definitive management have been described for trigger fingers in children. Unlike trigger thumb in which conservative management with splinting and passive range of motion showed a significant improvement in 48–86 % of cases and spontaneous resolution in 50 % of cases [1,6], trigger finger needs detailed workup for underlying conditions and ultimately necessitates surgical release [6]. In this study, we report a rare case with bilateral index and ring fingers triggering in a 5-year-old boy, with a brief literature review on its incidence, pathophysiology, and management preferences. This case report has been reported in line with the SCARE 2025 criteria [7].

## 2 Presentation of case

This is a 5-year-old boy, medically free, has no known allergies or prior admissions, who came to our clinic with his mother. She reported on and off bilateral triggering of index and ring fingers that is passively correctable for 1 year. Examination showed palpable nodule over the index and ring fingers bilaterally and a passively correctable trigger (Video 1). Patient referred to physiotherapy where he started splinting and specific exercises for 4 months with no improvement, so the patient was booked for operating room to do left index and ring finger annular pulleys release. Intraoperatively, there was no abnormal findings or obvious nodules apart of thickening of an annular pulley, the tendon glided freely after the release, and there was no residual triggering. 3 months after the first procedure, we did the same for the right hand. Patient seen in the clinic after 3 months with full range of motion for bilateral hands, completely healed scars, and no more triggering (Video 2).

## 3 Discussion

Trigger finger is extremely rare in pediatrics, and its bilateral occurrence is the rarest. It is considered difficult to treat because of its infrequency and ambiguous pathophysiology. First, a thorough diagnosis must be made, any underlying conditions must be ruled out, and then conservative vs. surgical treatment must be initiated [2].

Multiple risk factors for trigger fingers exist, including anatomical variability, trauma, infection, and some underlying conditions such as diabetes and mucopolysaccharidosis [[2], [3], [4]]. Four case reports are published in the literature, of which two are associated with a viral infection and one of them was diagnosed in a patient with neurofibromatosis type I [5,8]. Our patient was a medically free male who had an unremarkable history of trauma or signs of viral infections before the presentation, all of which have been associated with the diagnosis of trigger fingers in the pediatric population [[2], [3], [4], [5],^8^].

It is still unclear if it is a congenital or acquired phenomenon, but many authors believe it is an acquired condition. Jia et al. observed that symptoms of trigger finger in pediatrics appear later than those of trigger thumb [3]. Unlike our case, it is suggested by Sharma et al. that the mean age of its appearance is 11 months; however, three case studies reported its occurrence at ages of three, four, and seven years [2,4,5,8]. With this report, we add to the literature a case of bilateral multiple trigger fingers at the age of 5 years.

Pathophysiology of trigger fingers varies greatly between children and adults. Although A1 pulley hypertrophy and tendon sheath thickening are more pronounced in adults, thickening of tendons and nodule formation is greatly observed in children more than in adults [3]. In addition, the anatomical abnormality differs between thumb and other digits triggering in children. As illustrated by the reported sources, triggering of digits may result from FDS and FDP abnormal relationship and adhesions, formation of tendon nodules, and narrowness of distal annular pulleys [1,2]. On the other hand, the triggering of thumb is significantly associated with FPL thickening or tightness of the flexor tendon sheath or both [1]. As a result, a simple release of the A1 pulley as in thumb and adult triggering is usually insufficient to release the trigger finger, additional exploration, release of the A2 or A3 pulleys, or cutting a slip of FDS may be required [1,2]. Such as in our patient, after releasing the A1 pulley, partial A2 pulley release was needed to fully release the tendons and allow their smooth gliding.

After a thorough history taking and physical exam, a diagnostic work-up should be decided according to the suspected pathology [4]. If any underlying condition is suspected, its management is prioritized over the trigger finger. For example, if a recent viral infection is presumed, we may give the patient an observation period with follow-up waiting for symptom alleviation. As Sharma et al. published a pediatric case of bilateral trigger fingers associated with a viral infection, which was completely resolved once the viral infection resolved [8].

Unlike trigger thumb which is commonly self-limiting, the definitive management of trigger finger in children frequently requires surgical release [8]. Although it ultimately needs surgical management, some authors recommend starting initially with conservative management as splinting and range of motion exercises showed degrees of improvement [4,8]. In contrast to some reports that showed 30 % of patients treated conservatively either failed or ended up needing surgical release [4], Steenwerckx et al. reported two trigger finger cases that resolved spontaneously [8]. Our patient was referred to physiotherapy at the time of presentation, a trial of conservative management with splinting and range of motion exercises for 4 months failed, as a result, we decided to book the patient for the operating room and manage it surgically.

This case report presents a rare instance of bilateral multiple trigger fingers in a child, and its contribution to the limited literature is significant. The strength of this report lies in its careful clinical documentation, stepwise management plan, and postoperative follow-up. It is, however, a single case and hence has poor generalizability. Inadequate diagnostic imaging, and objective functional testing restrict conclusions regarding etiology and therapeutic response. Extended follow-up and further research are warranted to define better this rare disorder's natural history and optimal care.

## 4 Conclusion

In conclusion, unlike trigger thumb which is very common in pediatrics, trigger finger is considered rare and its bilateral hands involvement is the rarest. The pathophysiology of trigger finger in pediatrics differs that in adults, so detailed history, physical exam, and work-up must be done before any surgical intervention plan as it may be resolved spontaneously after managing the underlying cause.

The following are the supplementary data related to this article.

Video 1 A pre-operative video demonstrating the right-hand index and ring finger triggering. Video 1

Video 2 A post-operative video demonstrating resolution of right-hand index and ring finger triggering with full range of motion and with out deformity. Video 2

## Author contribution statement

All Authors participated equally in the design of the case report, read, and approved the final manuscript.

Maha Hanawi- literature search and writing of the article.

Rakan Alelyani- literature search, data collection, and contributing to the submission process.

Kayan Alotaibi- literature search and contributing to the submission process.

Abdulrahman Alhadlaq- writing the case presentation and conceptualization.

Ovais Habib- supervision and editing of the article.

Bandar Alaithan- supervision and review the article.

## Consent for Publication

Written informed consent was obtained from the patient's mother for publication of this case report. A copy of the written consent is available for review by the Editor-in-Chief of this journal on request.

## Ethical approval

This case report did not require approval from an ethical committee.

## Guarantor

Maha D. **Hanawi**

## Research registration number

Not applicable.

## Provenance and peer review

Not commissioned, externally peer reviewed.

## Financial disclosure statement

All authors have declared that they have no financial relationships at present or within the previous three years with any organizations that might have an interest in the submitted work.

## Declaration of competing interest

Authors have no potential competing interests or conflicts to report.
